# Head and neck myofibroma: A case series of 16 cases and literature review

**DOI:** 10.4317/medoral.26673

**Published:** 2024-08-01

**Authors:** Lucas Lacerda de Souza, Felipe Paiva Fonseca, Cinthia Veronica Bardález López de Cáceres, Ciro Dantas Soares, Alberto da Costa Gurgel, Hélder Antônio Rebelo Pontes, Flávia Sirotheau Corrêa Pontes, Carolina Almeida Paradela, Ivan José Correia-Neto, Yuri Kalinin, Marcio Ajudarte Lopes, Alan Roger Santos-Silva, Oslei Paes de Almeida, Pablo Agustin Vargas, Liam Robinson, Willie F P van-Heerden

**Affiliations:** 1 Oral Diagnosis Department, Piracicaba Dental School, University of Campinas, Piracicaba, Brazil; 2 Department of Oral Surgery and Pathology, School of Dentistry, Universidade Federal de Minas Gerais, Belo Horizonte, Brazil; 3 Department of Oral and Maxillofacial Pathology, School of Dentistry, Faculty of Health Sciences, University of Pretoria, Pretoria, South Africa; 4 Department of Pathology, Getulio Sales Diagnósticos, Natal, Rio Grande do Norte, Brazil; 5 Service of Oral Pathology, João de Barros Barreto University Hospital, Federal University of Pará, Pará, Brazil; 6 City Hall of the Seaside Resort of Praia Grande, São Paulo, Brazil; 7 PathCare Private Pathology Laboratory, Pretoria, South Africa

## Abstract

**Background:**

This study aimed to explore the clinical, histopathologic, and immunohistochemical characteristics of myofibromas (MFs) affecting the head and neck region.

**Material and Methods:**

Formalin-fixed paraffin-embedded tissue blocks of patients diagnosed with MFs in the head and neck were retrieved from the archives of three oral and maxillofacial pathology laboratories. Data including clinical, radiographic, microscopic and immunohistochemical findings, treatment employed, and follow-up status were retrieved from the patient's medical records or pathology reports.

**Results:**

Sixteen cases were included in the study. Females were slightly more affected than males. The first and second decades of life were more prevalent. The most common locations were the alveolar ridge and cheek. Although rare, some of the patients were presented with intraosseous lesions. Microscopically, tumors consisted of plump, spindle-shaped myofibroblasts arranged in whorls or fascicles with varying degrees of differentiation. Immunohistochemically, diffuse positivity for vimentin and α-SMA was observed, while Ki-67 mostly showed low immunoreactivity (<5%). Treatment primarily involved complete excision.

**Conclusions:**

MFs in the head and neck region are rare and predominantly affect female patients during the second decade of life. Despite their rarity, central MFs should be considered in the differential diagnosis of intraosseous lesions in infants.

** Key words:**Oral and maxillofacial pathology, soft tissue pathology, myofibroma, jawbones.

## Introduction

Myofibroblastic tumors, arising from myocytes or myoblasts, comprise a diverse group of soft tissue neoplasms (1,2). The World Health Organization (WHO) categorizes these tumors based on morphological features and aberrant gene product expression (1,3). Histologically, they exhibit neoplastic cells resembling myofibroblasts, expressing markers such as α-SMA and desmin (2-4). They are classified into four groups based on aggressiveness: benign, intermediate (either locally aggressive or with low metastatic potential), and malignant (1,5,6). Some benign variants may present atypical features, increasing the risk of sarcoma misdiagnosis (7).

Myofibroma (MF) is a benign mesenchymal tumor characterized by eosinophilic spindle-shaped cells arranged in fascicles, often surrounding blood vessels (8-10). MFs are the predominant myofibroblastic tumors in infants and children, but they may also manifest in adults (11,12). They typically present as painless solitary masses, predominantly affecting the skin and subcutaneous tissues in the head and neck region of infants (11,13). However, the trunk, extremities, and skeletal sites are not exempt (8,12).

Spontaneous regression of MFs is noteworthy, and post-excision local recurrences are infrequent (14). Both autosomal recessive and dominant inheritance patterns are documented (8,11,12). Intrauterine exposure to maternal estrogen is hypothesized to influence MF development, aligning with its frequent congenital presentation and subsequent regression post-estrogen exposure discontinuation post-birth (11,15). The designation "myofibromatosis" (MMF) applies to cases with multiple myofibroblastic lesions. Individual nodules resemble MFs, but their occurrence spans the dermis, subcutis, soft tissue, muscle, bone, and internal organs (16,17,18).

MFs within the head and neck region are atypical, predominantly involving the dermis and subcutis. Intra-osseous jawbone MFs are particularly rare, exhibiting a marked predilection for the mandible. Their presentation spans a broad age range, with a substantial proportion emerging within the first decade. Some literature suggests that up to 90% of cases manifest before age two (19). Owing to its rarity, clinical ambiguity, and histologic signatures, there is a heightened risk of misdiagnosing this lesion as a malignant or aggressive spindle cell neoplasm (11,15,18).

Most cases affecting the head and neck are reported as single case reports, and detailed analyses of cases are limited. This study investigated the clinical, microscopic, and immunohistochemical features of MFs affecting the head and neck.

## Material and Methods

- Study design

Cases diagnosed as MFs affecting the head and neck were retrospectively retrieved from the pathology archives of the University of Pretoria - Department of Oral Maxillofacial Pathology (Pretoria, SA), Laboratory Getulio Sales Diagnostics (Natal, Brazil), and University Hospital João de Barros Brreto (Belém, Brazil) during the period 2002 to 2022. Formalin-fixed paraffin-embedded (FFPE) tissue blocks were obtained, and new 4μm histological sections were cut and stained with hematoxylin and eosin (H&E) for microscopic description and diagnostic confirmation following the 5th edition of the World Health Organization Classification of Head and Neck Tumors of Soft Tissue Tumors (4).

Clinical features were retrieved from the patients' pathology reports or medical files. They included age, sex, tumor location, clinical presentation, time of evolution, imaging features, treatment employed, status at last follow-up (dead or alive), and follow-up time, if available.

- Immunohistochemistry

Immunohistochemistry (IHC) was performed on 3µm sections of FFPE tissues, which were first dewaxed with xylene and then hydrated in a descending ethanol series. The endogenous peroxidase activity was blocked with 10% hydrogen peroxide in a single bath for 15 minutes. After washing in PBS buffer (pH 7.4), the sections were incubated for 2 hours with primary antibodies and then exposed to high-sensitive horseradish peroxidase reagents (ADVANCE, Dako, Carpinteria, CA, USA) and diaminobenzidine tetrahydrochloride (DAB, Sigma-Aldrich, St Louis, MO, USA). The slides were counterstained with Carazzi hematoxylin for 3 minutes. Positive controls were used for each antibody, while the negative control was obtained by omitting the primary antibody.

Cases were submitted to the following primary antibodies: Vimentin, α-smooth muscle actin, and H-caldesmon. In cases with "borderline" histopathologic findings, the proliferation marker Ki-67 was used to differentiate from low-grade malignant tumors. Additional immunohistochemical markers were also used to screen for other possible myofibroblastic lesions, including desmin, S100, CD34, CD246 (ALK-1), and β-catenin (Supplement 1). The slides were digitally scanned using the Aperio ScanScope slide scanner CS® (Aperio Technology, Vista, CA, USA).

- Statistical analysis

Clinical, microscopic, and immunohistochemical features were descriptively analyzed. SPSS v22.0 (IBM, Germany) was employed for rigorous statistical analysis, ensuring data precision and authenticity.

## Results

- Demographic characteristics

Twenty cases diagnosed as MFs were initially retrieved from the assessed pathology archives; however, four cases were excluded due to a lack of clinical information to confirm the location of the neoplasm or an absence of histological sections or FFPE tissue blocks to confirm the diagnosis. Three cases were reclassified as myopericytoma, and one case was reclassified as desmoplastic fibroma. Therefore, 16 cases remained in the current study for analysis.

Table 1 details the clinicopathologic features of the included cases. Females were slightly more affected than males (male: female ratio of 1:1.28). Most patients were diagnosed in the first and second decades of life (10 cases; 62.5%) at a mean age of 20.4 years old (range of 1-67 years old). The majority of cases affected the alveolar ridge (03 cases; 18.75%), cheek (03 cases; 18.75%), gingiva (02 cases; 12.5%), and mandible (02 cases; 12.5%). In addition, cases located in the floor of the mouth, submental region, neck, submandibular region, tongue, and maxilla were also evidenced (01 case per subsite; 6.25%).

- Clinical findings

**Figure 1:**
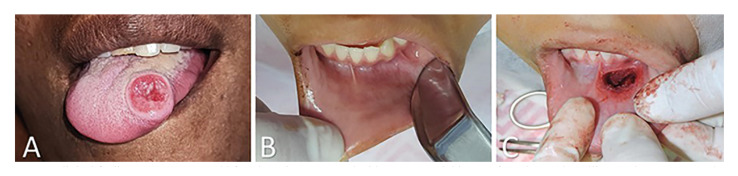
Clinical findings. A) A 37-year-old female patient presented with a one-month history of an ulcerated swelling on the tongue. B) The intraoral aspect of the same patient with a slight color alteration in the buccal mucosa. C) Transurgical procedure of the same patient showed total excision of the lesion.

**Figure 2:**
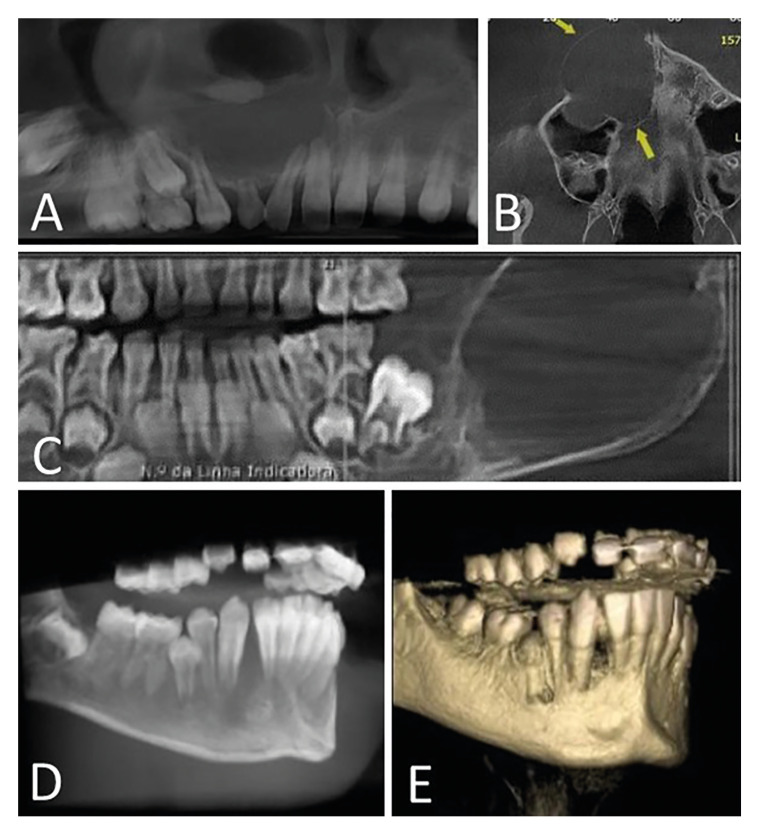
Radiographic aspect. A) CT image of a 14-year-old female patient revealed an expansive lesion with well-delineated borders in the maxilla causing displacement of the tooth and root resorption. B) An axial CT image of the same case showing a lesion with thinning of the cortical bone due to bony expansion (yellow arrows). C) CT image of a 5-year-old female patient presenting with an expansive lesion in the posterior region of the mandible causing thinning of the cortical bone. D) CT image of an 11-year-old male patient with a destructive lesion in the anterior mandible, causing bone resorption near the mental foramen associated with associated paresthesia. E) 3D reconstruction CT scan of the same patient.

The clinical presentation of the cases included in this study are illustrated in Fig. 1. The lesions had a mean evolution time of 4.75 months (range of 1-7 months). Swelling was observed in all cases analyzed (16 cases; 100%) and ulceration in six cases (37.5%). Radiologically, the lesions appeared as expansive, destructive radiolucent lesions, with borders varying from well-defined to ill-defined, with associated root resorption and displacement of teeth (Fig. 2). Treatment information was provided in five cases, whereby complete excision was the treatment modality used in all cases (100%), with a mean follow-up period of seven months (range of 2-12 months). No patient demonstrated recurrence at last follow-up.

- Microscopic findings

**Figure 3:**
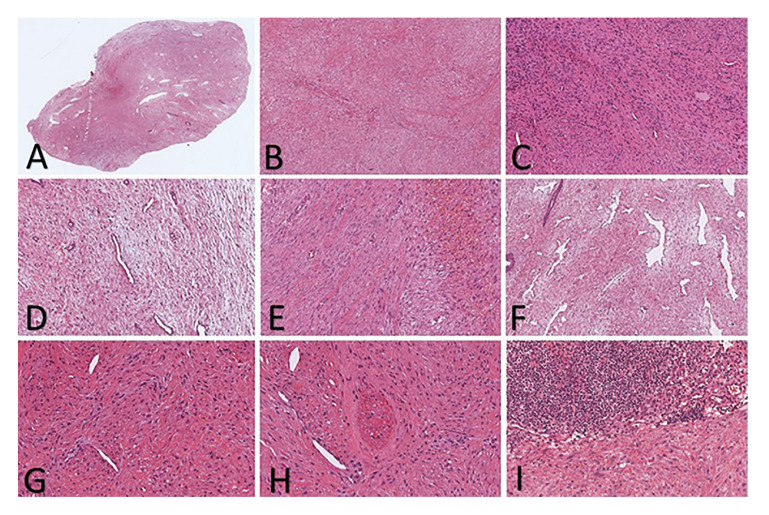
Histopathologic presentation. A) A fragment of tissue showing areas with different cellularity associated with vessels of different calibers (H&E, 5x). B) The cells were organized in either whorls or fascicles, exhibiting different levels of differentiation in a myxoid appearance (H&E, 10x). C) The lesion also presented cells with higher cellularity and hyperchromatic nuclei (H&E, 10x). D) Displaying various degrees of differentiation along with a myxoid appearance and the presence of blood vessels (H&E, 20x). E) The lesion also featured cells with increased cellularity, characterized by hyperchromatic nuclei and hemorrhage (H&E, 20x). F) The lesions showed thin-walled branching blood vessels resembling a hemangiopericytoma-like pattern (H&E, 10x). G) Atypical presentation of myofibroma demonstrating significant cellular pleomorphism with hyperchromatic nuclei (H&E, 40x). H) Occurrence of tumor infiltration in a perineural pattern in the atypical myofibromas (H&E, 40x). I) Occurrence of inflammatory infiltrate around the tumor (H&E, 40x).

Table 2 details the microscopic features of the included cases. The tumors were characterized by plump, spindle-shaped myofibroblasts arranged in whorls or fascicles, displaying varying degrees of differentiation. Centrally, the tumors appeared more hypercellular, while peripherally, they showed a less cellular, myxoid appearance. Interspersed throughout the tumor were thin-walled branching blood vessels surrounded by concentric layers of neoplastic cells, reminiscent of a so-called hemangiopericytoma-like vascular pattern. Cellular pleomorphism was observed in one case, and muscle/bone infiltration in three cases (18.75%). None of the cases displayed atypical mitotic Figures or tumor-associated necrosis. Fifteen cases (93.75%) presented with an unencapsulated, but well-circumscribed periphery (Fig. 3) (Table 2).

- Immunohistochemical results

**Figure 4:**
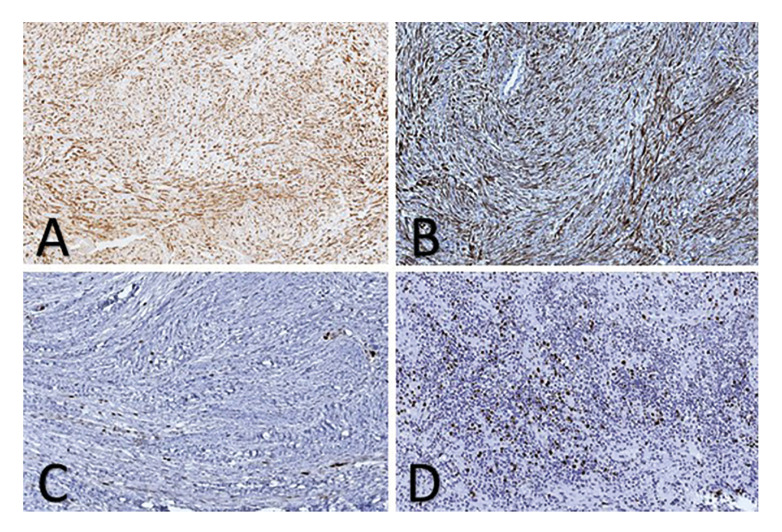
Immunohistochemical staining pattern. A) Cytoplasmic expression of vimentin (DAB, 20x). B) Cytoplasmic expression of α-SMA (DAB, 20x). C) Nuclear expression of Ki-67 <5% (DAB, 20x). D) Nuclear expression of Ki-67 around 10% in atypical myofibromas (DAB, 20x).

The analyzed cases showed positive staining for vimentin in thirteen cases (81.25%) (Fig. 4). All analyzed cases showed diffuse positivity for α-SMA, giving a so-called "Tram-track" pattern (Fig. 4). Ki-67 mostly showed immunoreactivity of less than 5% (14 cases; 87.5%) (Fig. 4), with one case classified as an atypical MF showing positivity of around 10% (6.25%) (Fig. 4). In one case, desmin was expressed focally (6.25%). All of the analyzed cases were negative for H-caldesmon, CD34, S-100, CD246 (ALK-1), and nuclear β-catenin (Table 2).

## Discussion

MFs represent the most common fibrous proliferation in infants, predominantly affecting the head and neck region (3,4,12). Over time, different terminologies, including congenital generalized fibromatosis, congenital multiple fibromatosis, multiple mesenchymal hamartomas, diffuse congenital fibromatosis, and multiple vascular leiomyomas of the newborn, have been used to describe the same entity (11,12). According to the WHO, solitary lesions are termed MF, while multicentric examples are labeled MMF (3,4). The current study presents a series of head and neck MFs, elucidating their clinical, histopathological, and immunohistochemical aspects.

The lesions typically manifest in patients during the first decade of life, consistent with the current research findings (20,21). This prevalence of postnatal lesions is commonly associated with multiple disseminated MFs throughout the body, known as MMF (18,21). However, despite this association, all cases in the current study presented as solitary lesions. Furthermore, the primary manifestation of MF in the head and neck region presents as firm and well-defined submucosal or subcutaneous masses (5,12). Within the oral cavity, the tongue, buccal mucosa, gingiva, and palate are the main anatomical sites affected (3,22,23). In the current study, the alveolar ridge and cheek emerged as the most frequently affected sites. Additionally, previous literature has noted a higher prevalence of lesions in females, consistent with the current study (3,19,24).

Central MFs of the jaws are exceedingly rare, with documented cases primarily observed in the mandible, although the current study reports a single case involving the maxilla (20,22). This variation of the tumor mainly occurs in adult patients, with males being more commonly affected (13,22). In contrast, the current study reported a predilection for females in the first and second decades of life. The most common clinical presentation is swelling, with some cases resulting in an oral mass due to cortical bone expansion. Larger tumors may cause symptoms such as pain, difficulty eating, and trismus. Some tumors are incidentally detected on routine radiographic exams. Radiologically, central MFs often appear as radiolucent, mostly unilocular, with well-circumscribed borders (13,20,22,25). While buccal cortical expansion and resorption of the cortical bone were evident in some of the presented cases, these are not typical features (22,25). In the radiologic differential diagnosis of mandibular lesions, central giant cell lesions and benign odontogenic or mesenchymal tumors should be considered (12,23).

In considering the histopathologic differential diagnosis of MF, a range of spindle cell lesions should be taken into account, including leiomyoma, neurofibroma, benign fibrous histiocytoma, solitary fibrous tumor, inflammatory myofibroblastic tumor, fibromatoses, infantile fibrosarcoma, leiomyosarcoma, hemangiopericytoma, nodular fasciitis, and desmoplastic fibroma (2,5). Histologically, MF manifests as a spindle cell neoplasm with a distinctive zonal appearance, characterized by elongated peripheral cells arranged in short fascicles or whorls and central round to polygonal-shaped cells forming around irregularly branching thin-walled blood vessels (26,27). Some cases may also present invasion of adjacent tissues and the occurrence of atypia or pleomorphism, consistent with case 7 from the current study (10). Diagnosing MF has become less challenging with the assistance of immunohistochemical markers. The myofibroblastic component typically exhibits positive staining for vimentin and alpha-smooth muscle actin, and is typically negative for S-100 protein and H-caldesmon, aiding in the exclusion of neural and smooth-muscle origin tumors (27,28). The absence of CD34 immunoexpression in MFs also aids in excluding solitary fibrous tumors from the differential list. Furthermore, the uniformly low cell proliferation index via Ki-67 IHC provides valuable information for excluding malignant mesenchymal neoplasms (2,9,20).

The biological behavior of MFs is still not fully understood. Typically, MF follows a benign clinical course, characterized by slow growth and often resolving completely after complete surgical removal (1,2,14). Some lesions have shown spontaneous regression, others have remained unchanged after partial removal, and a few even display rapid growth (19). Furthermore, the clinical course can vary depending on factors such as the number and location of the lesion, with genetics potentially playing a significant role (5,22,23). Considering the role of myofibroblasts in wound healing, previous trauma or injury may also contribute to forming these tumors (5,11).

Complete surgical excision is the primary treatment for both central and soft tissue MFs, boasting a notably low recurrence rate (12,19). In a comprehensive review, only a few oral MFs displayed recurrence after surgical intervention, while among patients with mandibular MFs, recurrence was rarely observed post-excision (12,14,19). Moreover, mandibular MFs typically lend themselves to easy removal from the surrounding bone tissue (19). Conversely, individuals with multicentric visceral lesions often face fatal outcomes either at birth or shortly thereafter due to complications like cardiopulmonary or gastrointestinal issues (16-18,28).

## Conclusions

In summary, MFs in the head and neck region are uncommon and tend to occur predominantly in female patients under the second decade of life. Integrating the clinical, histopathological, and immunohistochemical features is crucial for accurate diagnosis. Although exceedingly rare, central MFs should be considered in the differential diagnosis of well-circumscribed, unilocular lesions in infants.

## Figures and Tables

**Table 1:** Clinicopathologic findings of the analyzed cases.

Cases	Age	Sex	Site	Duration of symptoms (months)	Swelling	Ulcer	Necrosis	Treatment	Follow-up
1	36	F	Cheek	NR	Y	N	N	NA	NA
2	27	M	Gingiva	NR	Y	N	N	NA	NA
3	6	M	Gingiva	NR	Y	N	N	NA	NA
4	1	M	Cheek	NR	Y	Y	N	Total excision	NA
5	24	M	Floor of the mouth	NR	Y	N	N	NA	NA
6	53	M	Submental region	NR	Y	N	N	NA	NA
7	67	F	Alveolar ridge	NR	Y	Y	N	NA	NA
8	4	F	Neck	7	Y	N	N	NA	NA
9	11	F	Submandibular region	7	Y	N	N	NA	NA
10	37	F	Tongue	1	Y	Y	Y	NA	NA
11	14	F	Maxilla	NR	Y	Y	N	Total excision	6
12	5	F	Mandible	NR	Y	Y	N	NA	NA
13	11	M	Alveolar ridge	NR	Y	N	N	NA	NA
14	9	M	Alveolar ridge	NR	Y	Y	N	Total excision	2
15	10	F	Mandible	NR	Y	N	N	Total excision	8
16	12	F	Cheek	4	Y	N	N	Total excision	12

Abbreviations: M - Male; F - Female; NR - Not reported; Y - Yes; N - No; NA - Not available.

**Table 2:** Histopathologic and immunohistochemical findings of the studied cases.

Cases	Histopathologic analysis						Immunohistochemistry results								
	Diagnosis	Pleomorphism	Necrosis	Atypical mitotic figures	Muscle/ bone infiltration	Circumscribed (Y/N)	α- SMA	Ki67	Desmin	H- caldesmon	Vimentin	S100	CD34	ALK1	β-catenin
1	MF	No	No	No	No	Y	Pos	<5%	Neg	Neg	Pos	Neg	NA	NA	NA
2	MF	No	No	No	No	NA	Pos	<5%	Neg	Neg	Pos	Neg	NA	NA	NA
3	MF	No	No	No	No	Y	Pos	<5%	Neg	Neg	Pos	Neg	Neg	NA	NA
4	MF	No	No	No	No	Y	Pos	<5%	Neg	Neg	Pos	Neg	NA	Neg	NA
5	MF	No	No	No	No	Y	Pos	<5%	Neg	Neg	Pos	Neg	NA	NA	NA
6	MF	No	No	No	No	Y	Pos	<5%	Focal pos	Neg	NA	NA	Neg	NA	Neg
7	AMF	Yes	No	No	No	Y	Pos	10%	Neg	Neg	Pos	Neg	Neg	NA	NA
8	MF	No	No	No	No	Y	Pos	NA	Neg	Neg	NA	NA	NA	NA	NA
9	MF	No	No	No	No	Y	Pos	<5%	Neg	Neg	NA	Neg	Neg	NA	Neg
10	MF	No	No	No	No	Y	Pos	<5%	Neg	Neg	Pos	Neg	Neg	NA	NA
11	MF	No	No	No	No	Y	Pos	<5%	Neg	Neg	Pos	Neg	Neg	NA	NA
12	MF	No	No	No	No	Y	Pos	<5%	Neg	Neg	Pos	Neg	Neg	NA	NA
13	MF	No	No	No	No	Y	Pos	<5%	Neg	Neg	Pos	Neg	Neg	NA	NA
14	MF	No	No	No	No	Y	Pos	<5%	Neg	Neg	Pos	Neg	Neg	NA	NA
15	MF	No	No	No	No	Y	Pos	<5%	Neg	Neg	Pos	Neg	Neg	NA	NA
16	MF	No	No	No	No	Y	Pos	<5%	Neg	Neg	Pos	Neg	Neg	NA	NA

Abbreviations: MF - Myofibroma; AMF - Atypical myofibroma.
